# CAR-T therapy: pioneering a new era in the treatment of autoimmune diseases

**DOI:** 10.3389/fimmu.2025.1625166

**Published:** 2025-08-13

**Authors:** Yuanhao Wu, Luyao Han, Yu Wang, Mengjiao Gu, Yunuo Wang, Jingyue Gao, Hanjing Huang, Chen Li

**Affiliations:** ^1^ Department of Rheumatology, First Teaching Hospital of Tianjin University of Traditional Chinese Medicine, National Clinical Research Center for Chinese Medicine Acupuncture and Moxibustion, Tianjin, China; ^2^ Department of Dermatology, Tianjin Institute of Integrative Dermatology, Tianjin Academy of Traditional Chinese Medicine Affiliated Hospital, Tianjin, China

**Keywords:** chimeric antigen receptor T-cell therapy, CAR (chimeric antigen receptor), SLE - systemic lupus erythematosus, autoimmune diseases, adverse (side) effects

## Abstract

CAR-T therapy, an innovative immunotherapeutic approach, genetically modifies T cells to express CARs, enabling targeted destruction of specific antigen-expressing cells. Initially developed for oncology, CAR-T therapy has shown significant potential in treating autoimmune diseases. By targeting CD19+ B cells, CAR-T therapy has demonstrated rapid and sustained remission in refractory cases, with studies showing normalized laboratory parameters and reduced disease activity. At the same time, CAR-NK, CAAR-T and CAR-Treg technologies further broaden therapeutic strategies. However, some adverse effects also exist, including CRS, ICANS and so on. Despite these challenges, CAR therapy represents a promising advancement in autoimmune disease treatment, with ongoing research aimed at enhancing efficacy, durability, and safety. Continuous innovation is essential to address limitations and optimize therapeutic outcomes.

## 1 The application of CAR-T therapy in autoimmune diseases

**Figure 1:**
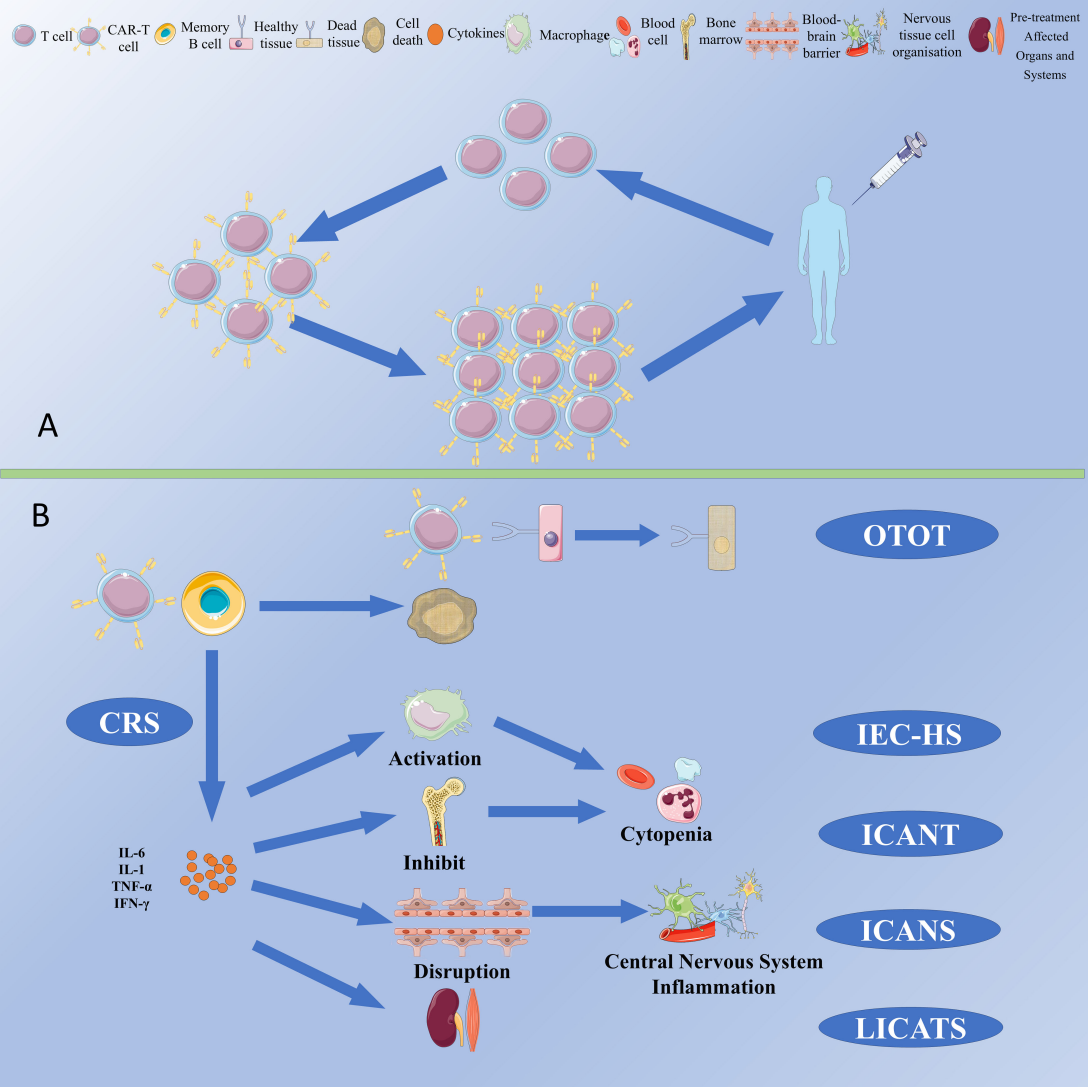
**(A)** Schematic for manufacturing and administration workflow of CAR-T Therapy. **(B)** CAR-T cell-associated toxicities.

Chimeric Antigen Receptor T-cell (CAR-T) therapy represents an innovative immunotherapeutic approach wherein T lymphocytes are genetically modified to express chimeric antigen receptors (CARs), thereby enabling the precise recognition and targeted elimination of specific antigen-expressing cells (**Figure 1A**). Kuwana first reported the concept of chimeric receptors in 1987 (1). This therapy has demonstrated significant efficacy in oncology (2–4). With a deep understanding of the technology, it has gradually been applied to treat autoimmune diseases (**Table 1**). Conventional therapies for autoimmune diseases exhibit certain limitations, whereas CAR-T therapy offers a new strategy. However, the potential adverse effects associated with this treatment require consideration.

**Table 1:** Summary of AIDs cases treated with CAR-T therapy.

Author	Patients n	Diseases	Follow-up(mo)	Dose × 10^6^/kg	Treatments	Treatment after CAR-T	Target	AD
Zhang W et al. (5)	1	SLE/ DLBCL	23	5.3	R-CHOP	–	CD19-BCMA	–
Mougiakakos D et al. (6)	1	SLE	1.5	1.1	HCQ/GC/CTX/MMF/TAC/BEL/RIX	Low-dose GC	CD19	–
Mackensen A et al. (7)	5	SLE	17	1	P1:GC/HCQ/MMF/CTX/TAC/BEL/RIX P2/P3:GC/HCQ/MMF/CTX/BEL P4:GC/HCQ/MMF/AZA/MTX/LEF/BEL P5:GC/HCQ/MMF/AZA/BEL	–	CD19	P2/P3/P4 developed CRS(Grade 1) and P4 used tocilizumab to treat.
Li M et al. (8)	1	SLE	6	0.5	GC/HCQ/IVIG/TAC/CsA/SRL/RIX/Danazol/Eltrombopag/rhTPO	Low-dose GC(5mg)/HCQ	CD19	CRS(Grade 1) (physical cooling methods)
Taubmann J et al. (9)	1	SLE	3	–	GC/HCQ/MMF/TAC/CTX/BEL/RIX	–	CD19	–
Krickau T et al. (10)	1	SLE	6	1	GC/HCQ/AZA/MMF/BEL	–	CD19(4-1BB)	CRS(Grade 1)*; lymphodepletionassociated;pre-existing anaemia
Müller F et al. (11)	8	SLE	29	–	P1:GC/HCQ/MMF/CTX/TAC/RIX/BEL P2/P3:GC/HCQ/MMF/CTX/BEL P4:GC/HCQ/MMF/MTX/AZA/BEL/LEF P5:GC/HCQ/MMF/AZA/BEL P6:GC/HCQ/MMF/MTX/AZA/CTX/TAC/BEL/RIX/LEF/Bortezomib/Upadacitinib/Immunoadsorption/UST P7:GC/HCQ/MMF/CTX/TAC/RIX/BEL P8:GC/HCQ/MMF/MTX/AZA/RIX/BEL/ECP/Lenalidomide/Thalidomide/UST/IL-2i	–	CD19(4-1BB)	P2/P3/P4/P6/P8 developed CRS(Grade 1)
	4	SSc	13	–	P1:MMF/MTX P2:GC/MMF/MTX P3:MMF/MTX/RIX/TCZ P4:MMF/CTX/Nintedanib	–	CD19(4-1BB)	P1/P2/P3 developed CRS(Grade 1)
	3	IIM		–	P1:GC/CTX/TAC/RIX/IVIG P2:GC/MMF/CTX/TAC/RIX/IVIG/T/TCZ/Ocrelizumab P3:GC/CTX/RIX/IVIG/Ocrelizumab		CD19(4-1BB)	P1/P2developed CRS(Grade 1);P3 developed CRS(Grade 2)
Wang W et al. (12)	13	SLE	48	3	P1/P2:GC/HCQ/CTX/MTX P3:GC/HCQ/MMF/BEL/TAC P4:GC/HCQ/CTX/BEL P5:GC/HCQ/MMF/BEL P6:GC/HCQ/MMF/CTX/Thalidomide P7:GC/HCQ/MMF/CTX/TAC P8:GC/HCQ/MMF/CTX/BEL P9:GC/HCQ/MTX/Thalidomide P10:GC/HCQ/MMF/TAC P11:GC/HCQ/MMF/CTX/BEL/Tetacep P12:GC/HCQ/MMF/BEL/TAC P13:GC/HCQ/CTX/BEL		CD19-BCMA	There were 8 patients developed CRS(Grade 1) in this study and resolved with supportive care.
Jinhui Shu et al. (13)	9	SLE	8	25/50/75/100	P1:GC/HCQ/TAC/CsA P2:GC/HCQ/TAC/BEL/Telitacicept P3:GC/HCQ/MMF/Telitacicept P4:GC/HCQ/MMF/TAC/CsA P5:GC/HCQ/MMF/CTX/TAC P6:GC/HCQ/MMF/CTX/TAC/Telitacicept P7:GC/HCQ/MMF/CTX/CsA/BEL P8:GC/HCQ/MMF/CTX	–	CD19	There were 6 patients developed CRS(Grade 1);P2 developed CRS(Grade 2);All patients developed cytopenia;There were 7 patients developed hypogammaglobulinemia
Nicolai R et al. (14)	1	JDM	6	1	GC/MTX/CsA/MMF/HCQ/IVIG/CTX/RIX	–	CD19(4-1BB)	CRS(Grade 1);transient ane- mia (grade 2);neutropenia (grade 4)
Qin C et al. (15)	1	SRP-IMNM	18	0.93	GC/TAC/MTX/MF/CsA/CTX/PE/IVIG/MSCs infusion/TCZ/RIX	–	CD19-BCMA	CRS(Grade 1);transient cytopenia
Pecher AC et al. (16)	1	ASS	9	1.23	GC/MTX/LEF/AZA/RIX/Baricitinib/IVIG	AZA/MMF	CD19	CRS(Grade 1), ICAHT
Taubmann J et al. (17)	1	ASS	5	1	GC/CTX/MMF/TAC/IVIG/T/TCZ/RIX/Ocrelizumab	–	CD19	CRS(Grade 1)*; ICAHS(Grade 1) (DXM)
Müller F et al. (18)	1	ASS	6	1	GC/TAC/CTX/IVIG/RIX	–	CD19	CRS(Grade 1)*; hypogammaglobulinaemia
Wang X et al. (19)	2	SSc	6	1	P1:GC/CTX/HCQ/MMF/TCZ/BEL/telitacicept/Rapamycin/PE/MSCs infusion P2:GC/CTX/MMF/TCZ	–	Allogeneic CD19	–
	1	SRP-IMNM	6	1	GC/CsA/MTX/IVIG/CTX/HCQ/MMF/TAC/AZA/CsA/IVIG/TCZ/Abatacept	–	as abo	–
Bergmann C et al. (20)	1	SSc	6	1	MTX/MMF	–	CD19	–
Merkt W et al. (21)	1	SSc-ILD	6	5	CTX/MMF/Nintedanib	MMF/Nintedanib	CD19(4-1BB)	–
Haghikia A et al. (22)	1	RA;MG	5	1	GC/AZA/IVIG/RIX/Eculizumab	GC(4mg/day)/AChEi	CD19	–
Szabo D et al. (23)	1	RA;DLBCL	6	–	GC/HCQ/MTX/TCZ	–	CD19-CD20(4-1BB)	–
Li Y et al. (24)	3	RA	6	1	P1:GC/MTX/LEF/HCQ/ETN/ADA/T P2:GC/MTX/IGU/ETN/Baricitinib P3:GC/MTX/LEF/HCQ/IGU/Baricitinib/Abatacept	P1/P3 HCQ	CD19/aIL-6/aTNFα	–
Andre´ s Parı´s-Mun˜ oz et al. (25)	1	MDA5+DM-(RPILDR)	11	1	GC/TAC/MMF/Ruxolitinib/T/IVIG/PE/RIX/Anakinra/Emapalumab	–	CD19	–
Song-yun Wang et al. (26)	1	DLBCL/psoriasis	12	2	R-CHOP		CD19	CRS(Grade 1)
Ao Zhang et al. (27)	1	pro-B ALL/plaque psoriasis	3	2	–	–	CD19	CRS(Grade 1)

BEL, Belimumab; RIX, Rituximab; ETN, Etanercept; ADA, Adalimumab; T, Tofacitinib; IGU, Iguratimod; SRL, Sirolimus; UST, Ustekinumab; ECP, extracorporeal photopheresis; TCZ, Tocilizumab; PE, Plasma exchange; MSCs, Mesenchymal stem cell infusion; AChEi, acetylcholinesterase inhibitors.

*Use tocilizumab to treat.

### 1.1 Systemic lupus erythematosus 

**Figure 2:**
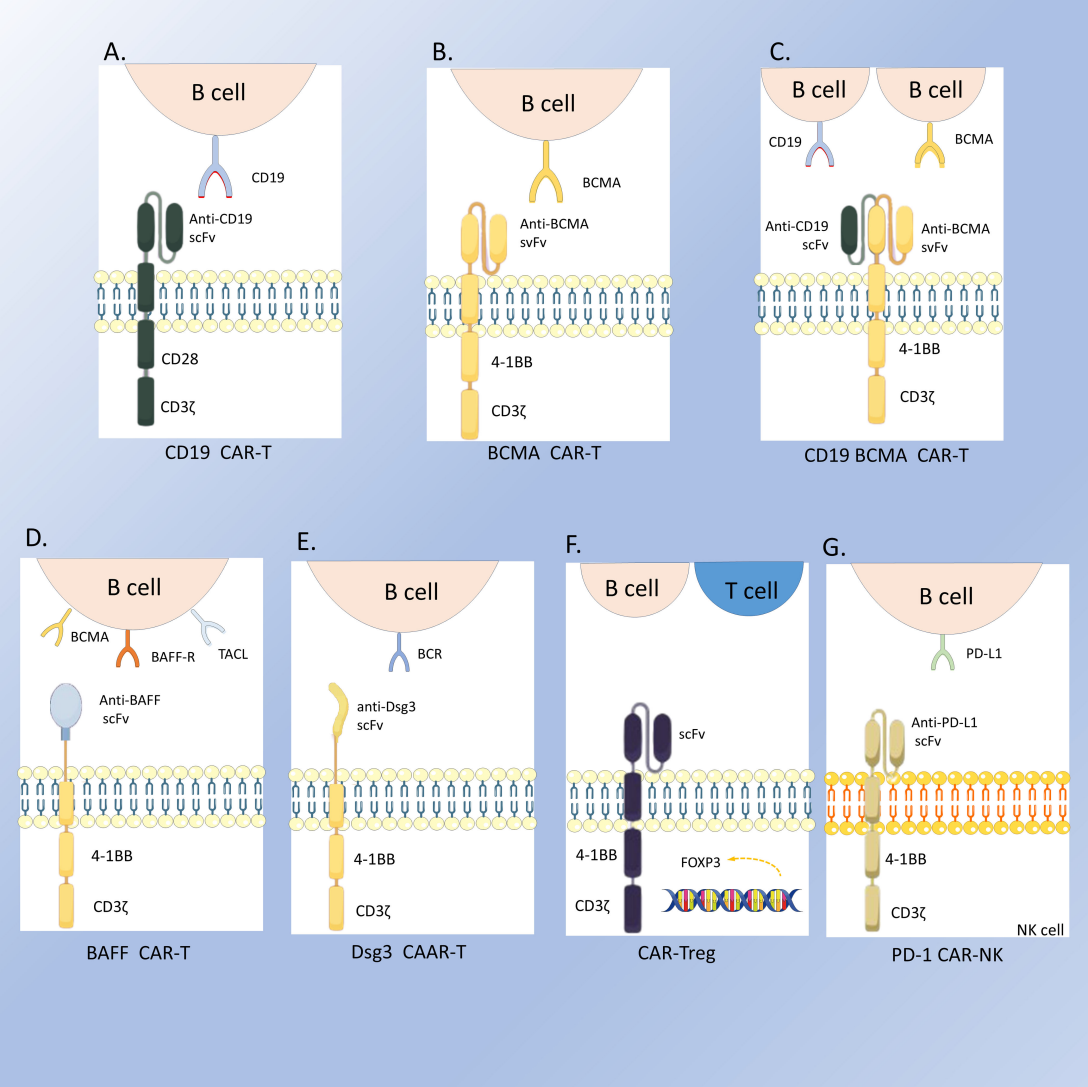
The structures of the CARs. **(A)** CD19 CAR-T; **(B)** BCMA CAR-T; **(C)** CD19-BCMA CAR-T; **(D)** BAFF CAR-T; **(E)** Dsg3 CAAR-T; **(F)** CAR-Treg; **(G)** PD-1 CAR-NK.

The pathogenesis of SLE is centered on the dysregulation of autoreactive B cells, which are abnormally activated and continuously produce antibodies targeting self-tissues, leading to inflammatory responses and tissue damage. Current B cell-targeted therapeutic strategies, such as rituximab and belimumab, have demonstrated some efficacy; however, some patients respond poorly to these treatments. In contrast, CAR-T therapy offers a more rapid and sustained targeting effect, providing a novel and promising option for SLE. CD19 is highly expressed at all stages of B cell differentiation and is considered a promising target for more effective and durable therapeutic responses in treating SLE (28, 29). Kansal (30) demonstrated that CD19-targeted CAR-T cells (**Figure 2A**) exerted therapeutic effects in a murine lupus model by depleting B cells and could prevent disease progression before the onset of illness. Subsequently, Jin (31) developed murine anti-CD19 CAR-T cells incorporating CD28 or 4-1BB co-stimulatory signals. They found that allogeneic CD19 CAR-T cell infusion prevented disease occurrence before symptom manifestation and provided therapeutic benefits in the later stages of the disease. CAR-NK therapy is another type of cell-based therapy that has also demonstrated potential for treating SLE. Specifically, anti-programmed death ligand 1 (PD-L1) CAR-NK 92 cells (**Figure 2B**) are capable of recognizing and eliminating follicular helper T cells with high expression of programmed death protein 1 (PD-1), thereby reducing the proliferation and differentiation of memory B cells, decreasing immunoglobulin secretion, and alleviating splenomegaly (32). Mougiakakos (6) first applied autologous anti-CD19 CAR-T therapy in patients with refractory SLE. Post-treatment, the patient’s abnormal laboratory parameters normalized, and the SLEDAI was reduced to 0. In their subsequent study, all five patients with refractory SLE achieved rapid and sustained remission (7). Zhang (5) reported a case of SLE with stage IV diffuse large B cell lymphoma (DLBCL) that achieved stable remission following anti-BCMA/CD19 CAR-T therapy (**Figure 2C**). Feng (33) also demonstrated effective and sustained remission in 12 patients with refractory SLE treated with anti-BCMA/CD19 CAR-T therapy. A study analyzing serum samples from six patients with refractory SLE before and after CAR-T therapy revealed decreased levels of IL-6 and TNF-α, accompanied by increased levels of IL-7 and BAFF, as well as a significant reduction in SLE-related antibodies (34). These findings all show CAR-T therapy’s potential in treating SLE.

### 1.2 Idiopathic inflammatory myopathies

CAR-T therapy has also demonstrated significant efficacy in the treatment of IIM, including juvenile dermatomyositis (JDM), antisynthetase syndrome (ASS), and immune-mediated necrotizing myopathy (IMNM). There are refractory ASS cases (16, 17) that were successfully treated with CAR-T therapy. Ann-Christin Pecher proposed that the cytokine release following CAR-T therapy may promote the expansion of CD8^+^ Temra cells, thereby sustaining inflammation. So, she administered mycophenolate mofetil one month after CAR-T treatment to induce durable clinical remission, thereby validating the hypothesis that B cells and T cells cross-activate to cause myositis. Rebecca Nicolai (14)reported a case of refractory JDM treated with CAR-T therapy, in which MRI at the 6-month follow-up showed resolution of lesions suggestive of myositis. This was accompanied by decreased expression of interferon (IFN) and IFN-induced chemokines, such as CXCL10 and CXCL9. Additionally, overexpression of IFN-α is crucial for maintaining B cell proliferation and stimulating the expansion of short-lived plasmablasts. IFN-λ promotes the differentiation of naïve B cells into plasmablasts via the mTORC1 pathway, leading to IL-6 and IL-10 and antibody production. SRP-IMNM is a particularly refractory subgroup of IMNM. Chuan Qin (15) reported using CAR-BCMA T therapy in a patient with refractory SRP-IMNM complicated by Sjögren’s syndrome. The patient exhibited sustained clinical and radiological improvement, with seroconversion of anti-SRP antibodies to negative. Additionally, within the T cell lineage, the highly cytotoxic and inflammatory CD8^+^ TE-1 cells were replaced by CD8^+^ TE-3 cells, indicating that CAR-T therapy may help modulate immune responses and reduce inflammation. Xiaobing Wang (19) also reported a case of refractory SRP-IMNM treated with allogeneic CD19 CAR-T therapy. The thigh muscle biopsy demonstrated a marked reduction in SRP expression and decreased B cell infiltration. Moreover, CAR-T therapy has also been proven effective and safe in severe pediatric cases. Andre’s París-Munoz (25) successfully treated a 10-year-old girl with MDA5+DM-rapidly progressive interstitial lung disease (RPILD) and macrophage activation syndrome (MAS), who had not responded well to conventional treatments. After receiving CD19 CAR-T therapy, the patient did not experience any serious adverse effects. For 11 months in an immunosuppressant-free context, the patient exhibited progressive improvements in motor and lung function, as well as restoration of the B cell compartment, without any autoimmune episodes.

### 1.3 Systemic sclerosis

In patients with SSc, the upregulation of B-cell activating factors and the abnormality of B-cell homeostasis make targeting CD19^+^ B cells a potential therapeutic strategy that could induce a more profound immune system reprogramming. Christina Bergmann (20) reported a case of a patient with SSc who received CAR-T therapy and experienced significant improvements in cardiac, joint, skin, and serological indicators. Wolfgang Merkt (21) reported a case of a patient with Scl-70 positive SSc treated with third-generation CD19 CAR-T therapy in combination with mycophenolate mofetil and nintedanib. Clinical evaluation at a five-month follow-up demonstrated a significant therapeutic response, characterized by reduced ANA titers and CRP levels, along with improved pulmonary function parameters and radiologic manifestations. Xiaobing Wang (19) reported the use of allogeneic CD19 CAR-T cells in two patients with relapsing diffuse cutaneous systemic sclerosis (dcSSc). Both patients exhibited robust responses to treatment, with skin biopsies revealing a significant reduction in B cell infiltration. These findings suggest that the depth of B cell depletion is far higher with CAR-T cell-based than antibody-based B cell depletion. Additionally, the patients exhibited potential reversal of tissue fibrosis, highlighting the therapeutic potential of this approach for treating systemic sclerosis.

### 1.4 Dermatology

Currently, CAR-T therapy is showing surprising effects in the treatment of psoriasis. Shaowei Qiu (27) successfully applied CD19 CAR-T therapy to treat a patient with high-risk pro-B acute lymphoblastic leukemia (pro-B ALL) complicated with severe plaque psoriasis. Notably, the patient’s psoriasis lesions completely resolved within 2 months after CAR-T infusion, and no signs of relapse were observed during the follow-up period. Huirui Wang (26) used CAR-T therapy to treat a 65-year-old male patient with a 45-year history of psoriasis and refractory DLBCL. After receiving CD19 CAR-T therapy, the patient not only achieved complete remission of refractory DLBCL but also experienced a significant improvement in his long-standing psoriasis, with only minimal residual lesions remaining on the neck. These findings suggest that CD19 CAR-T therapy may exert therapeutic effects on psoriasis by targeting B cells and modulating the immune microenvironment, offering a new research direction and potential therapeutic strategy for psoriasis treatment and highlighting the need for further exploration of the application and immunological mechanisms of CAR-T cell therapy in psoriasis.

### 1.5 Others

Beyond the aforementioned diseases, progress has also been made in other autoimmune conditions. For instance, a 37-year-old female patient with both Rheumatoid Arthritis (RA) and myasthenia gravis (MG) achieved complete remission of MG and a significant reduction in RA disease activity following CD19-CAR T therapy (22). In the treatment of RA, Bo Zhang (35) and colleagues combined universal anti-fluorescein isothiocyanate (FITC) CAR-T cells with FITC-T-labeled RA-T immunodominant peptides, thereby eliminating hybridoma cells from RA patients and RA patient-derived autoreactive B cell subsets. This approach offers a new direction for precise and customized treatment of RA based on the individual patient’s autoantigen profile. A study on pemphigus vulgaris reported that T cells engineered to express a chimeric autoantibody receptor (CAAR) (**Figure 2D**) exhibited specific cytotoxicity against cells expressing anti-Dsg3 BCRs *in vitro* and effectively eliminated these B cells *in vivo*. Cody D. Moorman (36) developed CAR-T cells and CAR-Tregs targeting X-C motif chemokine receptor 1 (XCR1) (**Figure 2E**) on the surface of conventional type 1 dendritic cells (DC1) to suppress experimental autoimmune encephalomyelitis (EAE). The results showed that both cell types modestly inhibited Th1-driven EAE. Dörte Lodka (37) first reported the efficacy of CD19 CAR-T cells in ANCA-associated vasculitis, demonstrating robust depletion of B cells and plasmablasts, a significant decline in MPO-ANCA levels, and protection of mice from ANCA-induced necrotizing crescentic glomerulonephritis (NCGN).

## 2 Adverse effects

Common cytotoxicities associated with CAR-T therapy include cytokine release syndrome (CRS), immune-cell-associated neurotoxicity syndrome (ICANS), immune effector cell-associated haematotoxicity (ICAHT), immune effector cell-associated haemophagocytic lymphohistiocytosis-like syndrome (IEC-HS), persistent B-cell aplasia, hypogammaglobulinemia (HGG), on-target off-tumor toxicity (OTOT) and local immune effector cell-associated toxicity syndrome (LICATS) (**Figure 1B**). Given the lower B cell burden in autoimmune diseases compared to that in neoplastic disorders, the incidence and severity of adverse effects tend to be correspondingly lower.

CRS is a systemic inflammatory syndrome mediated by the release of cytokines such as IL-6, with fever often being the primary clinical symptom (38). Most reported cases are mild and do not require medication; however, severe cases can be treated with tocilizumab, glucocorticoids, and other drugs (7). ICANS is initiated by the activation of immune cells, which release large amounts of inflammatory cytokines. This process can deplete mural cells (such as endothelial cells expressing CD19) and disrupt the blood-brain barrier, thereby activating microglia and manifesting as neurological symptoms (39). Corticosteroids are the first-line treatment for ICANS. ICAHT refers to bone marrow suppression and cytopenia after CAR-T therapy, which may be related to multiple factors such as target cell burden, hematopoietic reserve, and baseline inflammatory environment (40). After CAR-T therapy, most patients experience lymphopenia or neutropenia (grade 3 or 4), which usually recovers within 4 weeks. Thrombopoietin receptor agonists, glucocorticoids, and stem cell transplantation can improve cytopenia. OTOT is an adverse reaction caused by CAR-T cells attacking healthy tissues that express the same antigens as the pathological tissue. CAAR-T therapy, which targets and eliminates autoreactive cells secreting autoantibodies, offers a new strategy to reduce the risk of off-target effects (41). IEC-HS is a highly inflammatory syndrome resulting from macrophage activation, which has not been reported in CAR-T therapy for autoimmune diseases. Persistent CAR-T cell-induced B-cell hypoplasia and HGG are common complications of CAR-T therapy (11, 41), which may increase the risk of infection, mostly mild (42). To mitigate side effects and avoid unnecessary immune activation, the concept of on/off switches has been introduced into CAR-T therapy, enabling precise control over CAR-T cell activity and allowing them to remain quiescent during remission periods to reduce the risk of unnecessary immune activation and toxicity (43). In a recent study conducted in Germany, LICATS events were documented (44). The researchers posited that LICATS represents a form of toxicity distinct from CRS, characterized by localized and self-limiting organ dysfunction that correlates with the organs and systems affected prior to treatment. The occurrence of LICATS may be associated with the depletion of B cells in tissues by CAR T-cell therapy, thereby triggering localized inflammatory responses. Typically, LICATS can resolve spontaneously or with the administration of low-dose glucocorticoids. In clinical practice, it is crucial to differentiate LICATS from disease relapse or CRS to avert unwarranted therapeutic interventions.

## 3 Other CAR Therapy

CAR-NK cells are NK cells that have been genetically engineered to express chimeric antigen receptors (CARs) and have shown potential in the treatment of autoimmune diseases. Research by Seth D. Reighard (32) has demonstrated that CAR-NK cells derived from the NK-92 cell line and targeting PD-L1 successfully reduced the proliferation of memory B cells as well as the secretion and differentiation of immunoglobulins in a humanized lupus-like disease mouse model. Xiaobing Wang has (45) developed an off-the-shelf allogeneic iPSC-derived CD19/BCMA CAR-NK dual-targeting cell product, which has been successfully applied to a patient with refractory dcSSc. In addition, CAR-NK cells can secrete specific cytokines such as IFN-γ and GM-CSF, which help to reduce the risk of CRS and neurotoxicity (46). Compared with traditional CAR-T cells, CAR-NK cells offer more flexibility as they can eliminate target cells not only through antigen-dependent cytotoxicity but also through antigen-independent mechanisms (47).

CAAR T therapy is an innovative immunotherapy that incorporates autoantigens into the receptors of T cells, enabling these T cells to specifically recognize and eliminate autoreactive B cells. Jie Zhou (48) constructed GPIbα CAAR T cells, which demonstrated robust persistence and specific cytotoxicity in an NSG mouse model, significantly reducing the growth of anti-GPIbα hybridoma cells without apparent organ toxicity. When applied to immune thrombocytopenia (ITP) patients, these cells could identify and eliminate autoreactive B cells from the patients’ sera. GPIbα CAAR T therapy is a precision cellular immunotherapy with the potential to induce complete and durable remission in refractory and relapsed ITP patients. CAAR T therapy was also applied to the treatment of pemphigus and muscle-specific tyrosine kinase myasthenia gravis.

CAR-Treg cells are obtained by co-culturing T cells with two vectors containing the CAR gene and the FOXP3 gene. They can not only interact with dendritic cells to convert conventional T cells into induced Treg cells, thereby expanding the effect of immune tolerance (49), but also suppress immune responses targeting antigens and regulate other related immune responses through bystander suppression mechanisms (secreting anti-inflammatory cytokines such as IL-10 and TGF-β to modulate the local immune environment) (50). In the treatment of autoimmune diseases, CAR-Treg cells have been applied. Related studies have shown that in a 2,4,6-trinitrophenol (TNP)-induced IBD mouse model, TNP-CAR-Treg cells can be observed at the inflamed colon site. This indicates that CAR-Treg cells designed against specific antigens can alleviate colitis in mice by recognizing and suppressing overactive immune responses (51). Tenspolde (52) also found that insulin CAR-Treg cells showed effective control of type 1 diabetes in both *in vitro* and *in vivo* experiments.

With the continuous expansion of CAR technology, CAR-T cells targeting the BAFF ligand have shown unique advantages. Researchers used the non-viral transposon system TcBuster (TcB) to stably integrate BAFF-CAR into T cells (**Figure 2F**). This method is safer and more economical than traditional viral transduction methods, and these CAR-T cells can target three receptors: BAFF-R, BCMA, and TACI simultaneously (53). Studies have shown that BAFF CAR-T cells can specifically deplete autoreactive B cells by targeting BAFF receptors, thereby reducing the production of autoantibodies. This phenomenon has been confirmed in various lupus animal models, including humanized lupus mouse models and MRL/lpr mouse models (54). As research deepens, it is hoped that more types of CAR-T cells will be applied to the treatment of autoimmune diseases in the future, providing patients with more options.

CAR-T therapy has demonstrated the potential to alleviate symptoms and improve the quality of life for patients with autoimmune diseases. However, the sustainability and stability of its therapeutic effect still pose challenges. Because CAR-T therapy cannot eliminate the patient’s pathological T cells or some CD19^-^ plasma cells that produce antibodies, patients may experience persistent disease or recurrence after the treatment (55). It has been reported that a patient with IIM relapsed 9 months after CD19 CAR-T therapy. Reinfusion of CD19 CAR-T cells failed to expand. After the failure of the reinfusion of CD19 CAR-T cells, the patient then received BCMA CAR-T cell therapy (**Figure 2G**) and achieved stable drug-free remission (25). This suggests that BCMA targeting addresses autoreactive plasma cells resistant to CD19-directed therapies and switching CAR-T cell targets may be an effective strategy in the treatment of AIDs. Therefore, it is crucial to continuously innovate to address the limitations of CAR-T therapy in autoimmune diseases to enhance efficacy and durability and minimize adverse events. To further explore the potential of CAR-T therapy in autoimmune diseases, multiple registered clinical trials have been initiated worldwide (**Table 2**). Off-the-shelf allogeneic CAR-T therapy recently has been utilized in the treatment of autoimmune diseases with excellent therapeutic responses (19). This approach significantly reduces the treatment waiting time and demonstrates superior cost-effectiveness compared to conventional therapies. However, off-the-shelf CAR-T therapy also has disadvantages. For example, their expansion and persistence are not as good as those of autologous CAR-T cells. There is also the possibility of graft-versus-host disease(GVHD). At present, there are also studies (45) working on solving these problems, such as using iPSC-derived NK cell-based CAR therapies and knocking out certain genes. In summary, the emergence and continuous improvement of CAR-T cell therapy offers a promising and effective treatment option for patients with autoimmune diseases. It also faces many challenges and limitations that need to be solved.

**Table 2:** Summary of registered clinical trials of AIDs cases treated with CAR-T therapy.

Diease	Rregistry number	CAR-construct	Dose (CAR-T cells× 10^6^/kg)	Primary outcome measures
AIDs(SLE/SS/SSc/AAV/IIM/APS)	ChiCTR2400084552	CD19	–	DLT;Safety;Efficacy
AIDs(SLE/SSc)	ChiCTR2400092462	CD19	–	DLT;SRI-4
AIDs(SLE/RA/SS/SSc)	NCT06503224	CD19-BCMA	0.5-3× 10^6^/kg	Safety
AIDs(SLE/AAV/IIM)	NCT06462144	CD19/CD20	–	DLT;Safety
AIDs(SLE/SSc/AAV/IMM/SS)	NCT06548607	CD19	–	Safety
AIDs(SLE/SSc/AAV/IIM/NMOSD/MS/MG)	NCT06775912	CD19-BCMA	–	Safety
AIDs(SLE/SSc/SS/APS/IIM/AAV)	NCT06688799	CD19	–	DLT;AE;ORR
AIDs(SLE/SSc/IIM/AAV/SS/ITP/APS)	NCT06762119	CD19	–	DLT;Safety
AIDs(SLE/SSc/SS/APS/IIM/AAV)	NCT06680388	CD19	0.5× 10^6^/kg traditional “3 + 3” design	DLT
AIDs	NCT06661811	CD19	–	DLT;AE
AIDs(SLE/SSc/SS/APS/IIM/AAV)	NCT06685042	CD19	1× 10^6^/kg	AE;ORR
AIDs(SLE/IMNM/NMOSD/MS/MG)	NCT06249438	CD20-BCMA	–	DLT;AE
AIDs(SS/SLE/SSc/AAV/IIM)	NCT06056921	CD19	–	Safety;Efficacy
AIDs(SLE/SSc/IIM)	NCT05869955	CD19	–	AE;DLT;RP2D
AIDs(CD/DM/AOSD/RA)	NCT05239702	CD7	–	AE;DLT
AIDs(SLE/SSc/SS)	ChiCTR00049920	CD19-BCMA	–	AE;DLT
SLE	NCT05030779	CD19-BCMA	–	AE;DLT
SLE	NCT0661337	CD19-BCMA	–	Safety
SLE	NCT05765006	CD-19	15-150× 10^6^/kg	DLT;Determine RP2D
SLE	NCT06310811	CD-19	1/5× 10^6^/kg	DLT;Safety
SLE	NCT06297408	CD-19	–	DLT;Determine RP2D
SLE	NCT06038474	CD19-BCMA	–	Safety;Tolerability
SLE	NCT03030976	CD19(4-1BB)	1-10× 10^6^/kg	Safety
SLE	NCT06752876	CD19	–	Safety
SLE	NCT06711146	CD19	–	DCR;MTD
SLE	NCT06710717	CD19	–	Clinical remission;AE
cSLE	NCT06691152	CD19	1/3/6× 10^6^/kg	AE
SLE	NCT06681337	CD19-BCMA	–	AE
SLE	NCT06585514	CD19	–	ORR;DLT;AE
SLE	NCT05030779	CD19-BCMA	1-4× 10^6^/kg	DLT;AE
SLE	NCT06222853	CD19	0.3/1/3× 10^5^/kg	Safety
SLE	NCT06150651	CD19	1× 10^6^/kg	Safety
SLE	NCT06121297	CD19(4-1BB)	–	AE
SLE	NCT05988216	CD19	–	Safety
SLE	EUCTR2022-003137-19-DE	CD19	1× 10^6^/kg	AE;DLT;RP2D
SLE	NCT05474885	CD19-BCMA	–	AE
SLE	NCT05085418	CD19-BCMA	1-4× 10^6^/kg	AE;DLT
SLE	EUCTR2022-001796-14-DE	CD19-BCMA	–	AE
SLE	NCT05846347	CD19-BCMA	0.8-3.6× 10^5^/kg	AE;DLT
SLE	NCT05930314	CD19	0.25-0.5× 10^8^/kg	AE
SLE	NCT05938725	CD19	–	AE;DLT;Efficacy
refractory NMOSD	NCT06633042	CD19-BCMA	1-4× 10^6^/kg	DLT;Safety
RA	NCT06503237	CD19	1-6× 10^6^/kg	Safety
RA	NCT06201416	CAR-Treg	–	DLT;Safety
RA	NCT06475495	CD19	–	Safety;Efficacy
RJDM	NCT06686524	CD19	1/3/6× 10^7^/kg	AE;TIS;DAS
IIMs(DM/ASyS/IMNM/JIIM)	NCT06154252	CD19(4-1BB)	–	AE
SSc	NCT06328777	CD19(4-1BB)	–	AE
SSc	NCT05085444	CD19-BCMA	1-4× 10^6^/kg	AE;DLT
SS	NCT05085431	CD19-BCMA	1-4× 10^6^/kg	AE;DLT

ORR, objective response rate; DLT, dose-limiting toxicity; AE, adverse events; TIS, Total improvement score; DAS, Disease Activity Score; RP2D, Recommended Phase 2 Dose; DCR, Disease control rate; MTD, Maximal Tolerable Dose.
